# Characterization of the complete chloroplast genome sequence of *Gynostemma microspermum* (Cucurbitaceae)

**DOI:** 10.1080/23802359.2021.2007811

**Published:** 2021-12-10

**Authors:** Xiao Zhang, Xiaodan Chen, Hao Zhang, Yuemei Zhao, Miaomiao Ju, Guifang Zhao

**Affiliations:** a Key Laboratory of Resource Biology and Biotechnology in Western China (Ministry of Education), College of Life Science, Northwest University, Xi’an, China; b College of Life Sciences, Sun Yat-sen University, Guangzhou, China; c School of Biological Sciences, Guizhou Education University, Guiyang, China

**Keywords:** *Gynostemma microspermum*, chloroplast genome, illumina sequencing, phylogenetic relationship

## Abstract

*Gynostemma microspermum* C. Y. Wu et S. K. Chen is an endemic creeping herbaceous species mainly distributed in dense forests on limestone in northwestern China. Here, the complete chloroplast genome sequence of *G. microspermum* was obtained by Illumina pair-end sequencing. The circular complete chloroplast genome of *G. microspermum* is 158,692 bp in length and contains a large single copy region (87,452 bp), a small single copy region (19,068 bp) and two short inverted repeat regions (26,086 bp). The genome sequence encodes 133 genes including 87 protein-coding genes, 37 transfer RNA genes, 8 ribosomal RNA genes and 1 pseudogene. The maximum likelihood (ML) phylogeny estimation shows that *G. microspermum* is sister to all other analyzed species of the genus *Gynostemma* with high bootstrap support.

The genus *Gynostemma* Bl. belonging to family Cucurbitaceae consists of 17 creeping herbaceous species mainly distributed in east and southeast Asia. They are usually used as tea and considered as medical plants due to their anti-inflammatory properties, anticancer effects and weight controlling function (Xie et al. 2010). *Gynostemma microspermum* C. Y. Wu et S. K. Chen is an endemic species mainly distributed in dense forests on limestone in northwestern China at an altitude between 800 m and 1400 m (Chen 1995). Unfortunately, wild population of *G. microspermum* was severely decimated during the past years because of habitat destruction and urbanization, which could accelerate biodiversity declines and species extinctions (Ceballos et al. 2015). Therefore, more measures should be taken to protect the wild resources of *G. microspermum* urgently and ensure species diversity. Previous studies have shown that genetic and genomic researches would make contribution to species conservation. However, some studies focus on genetic relationships of *Gynostemma* species based on chloroplast genome or few gene fragments (Zhao et al. 2015; Zhang et al. 2017), but no genomic studies on *G. microspermum*. Thus, we assembled and characterized the complete chloroplast genome sequence of *G. microspermum* based on the Illumina pair-end sequencing. This study will provide a valuable complete chloroplast genomic resource and contribute to the further study on the phylogenetic analysis, systematic evolution and conservation genetics of *G. microspermum.*

Fresh and healthy leaves of *G. microspermum* were collected from adult plants in Mengla county (Yunnan, China; 21.74°N, 101.39°E), and a specimen was deposited at Northwest University (Xiao Zhang, zhxiaao@163.com) under the voucher number NWU020161211. Total genomic DNA (number: DNA202008230024) was extracted by CTAB method (Doyle 1987) using for high-throughput sequencing with the Illumina Hiseq 2500 platform by Genesky Biotechnologies Inc. (Shanghai, China). A total of 1.3 Gb raw reads were obtained with an average length of 149.9 bp yielding 1232.4× coverage of the genome. After quality-trimmed using the CLC Genomics Workbench v7.5 (CLC bio, Aarhus, Denmark) program, reference-guided assembly was performed twice to construct the chloroplast genome with the program MITObim v1.7 (Hahn et al. 2013) using published *Gynostemma pentaphyllum* (KX014626) and *Gynostemma cardiospermum* (KX852299) (Zhang et al. 2017) as references, respectively. Software Geneious R8 (Biomatters Ltd, Auckland, New Zealand) was used to annotate the complete chloroplast genome. Finally, the annotated chloroplast genome sequence of *G. microspermum* has been submitted to the GenBank with the accession number MZ286581.

The complete chloroplast genome of *G. microspermum* is 158,692 bp in length and contains a large single copy region (LSC, 87,452 bp), a small single copy region (SSC, 19,068 bp), and a pair of inverted repeat regions (IRa and IRb, 26,086 bp). The genome sequence encodes 133 genes including 87 protein-coding genes (CDS), 37 transfer RNA (tRNA) genes, 8 ribosomal RNA (rRNA) genes and 1 pseudogene. Among these annotated genes, 16 (*atpF*, *ndhA*, *ndhB*, *petB*, *petD*, *rpl2*, *rpl16*, *rpoC1*, *rps12*, *rps16*, *trnA-UGC*, *trnG-UCC*, *trnI-GAU*, *trnK-UUU*, *trnL-UAA*, and *trnV-UAC*) of them exist a single intron. Most of the genes occur in a single copy, but 8 protein-coding genes (*ndhB*, *rpl2*, *rpl23*, *ycf1*, *ycf2*, *rps7*, *rps12*, *orf70*), 7 tRNA genes (*trnA-UGC*, *trnI-CAU*, *trnI-GAU*, *trnL-CAA*, *trnN-GUU*, *trnR-ACG*, *trnV-GAC*) and 4 rRNA genes (*rrn16*, *rrn23*, *rrn4.5*, *rrn5*) occur in twice in IRa and IRb. The gene *infA*, which is proved to be a translation-related gene, is identified to be a pseudogene.

A total of 21 complete chloroplast genome sequences (Plader et al. 2007; Atherton et al. 2010; Rodriguez-Moreno et al. 2011; Sousa et al. 2016; Zhang et al. 2017, 2018a, 2018b) were selected to construct the phylogenetic relationships among the main representatives of Cucurbitales with *Corynocarpus laevigata* (HQ207704) as outgroup (Figure 1). The maximum likelihood (ML) phylogenetic analysis was performed using RAxML v7.2.8 (Stamatakis 2006) performed with 1000 replicates. The ML phylogeny estimation shows that *G. microspermum* is sister to all other analyzed species of the genus *Gynostemma* with high bootstrap support. This study on the complete chloroplast genome of *G. microspermum* would provide information to the demonstration of chloroplast genome structure and the understanding of its evolution.

**Figure 1.**
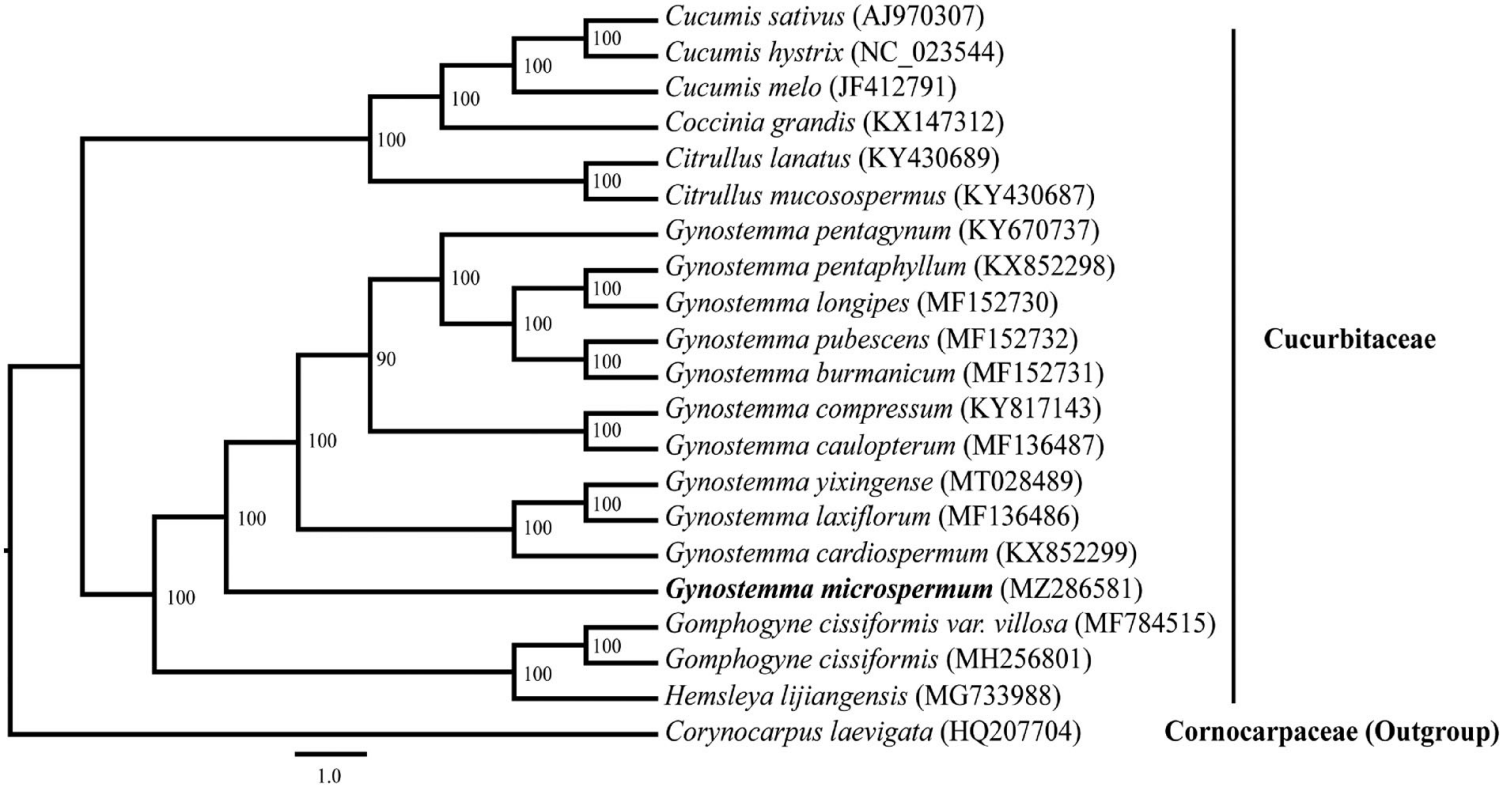
Phylogenetic tree based on 21 complete chloroplast genome sequences. The bootstrap values are indicated next to the branches. Accession numbers are listed together with each species.

## Data Availability

The genome sequence data that support the findings of this study are openly available in GenBank of NCBI at https://www.ncbi.nlm.nih.gov under the accession number MZ286581. The raw sequence data used in this research were deposited successfully with registered numbers of associated BioProject, Bio-Sample and SRA: PRJNA733705, SAMN19433685, and SRR14689372, respectively.
